# Calcineurin Inhibitors and Male Fertility in Renal Transplant Recipients: A Comprehensive Review of Molecular Pathomechanisms and Reproductive Toxicity of Cyclosporine A and Tacrolimus

**DOI:** 10.3390/ijms27198501

**Published:** 2026-09-23

**Authors:** Marta Grabowska, Małgorzata Piasecka

**Affiliations:** Department of Histology and Developmental Biology, Faculty of Health Sciences, Pomeranian Medical University, Żołnierska 48, 71-210 Szczecin, Poland

**Keywords:** testis, epididymis, prostate, calcineurin inhibitors, cyclosporine A, tacrolimus

## Abstract

This comprehensive review provides a detailed organ-resolved and translational synthesis of the effects of calcineurin inhibitors, such as cyclosporine A (CsA) and tacrolimus (FK506), on male reproductive function following kidney transplantation. This review summarises experimental and clinical evidence relating to the testes, epididymis, accessory glands, the hypothalamic–pituitary–gonadal axis and semen parameters. It also considers molecular mechanisms, clinical outcomes and transplant-related confounding factors. Experimental evidence indicates that calcineurin inhibitors can cause testicular injury in a dose- and duration-dependent manner, resulting in seminiferous epithelium disorganisation, impaired Sertoli–germ cell interactions, Leydig cell atrophy, reduced testosterone synthesis, and reduced sperm count and motility. In animal models, CsA appears to induce more pronounced histological and steroidogenic disruption. In contrast, murine studies suggest that FK506 primarily exerts molecular alterations on post-testicular sperm maturation. Proposed preclinical mechanisms include oxidative stress, mitochondrial dysfunction, apoptosis and microvascular remodelling. However, their direct role in human recipients remains to be fully verified. Clinical findings demonstrated that successful transplantation often improves pre-existing uremic hypogonadism, and semen quality usually recovers within one to two years, particularly with stable graft function and lower calcineurin inhibitor exposure. However, persistent abnormalities can occur, and outcomes are confounded by, among others, renal function or combination therapy. It is important to distinguish direct calcineurin inhibitor-induced reproductive toxicity from the confounding effects of chronic kidney disease, prior dialysis exposure, and glucocorticoid use, as these transplant-related factors significantly modulate gonadal function independently of calcineurin inhibition.

## 1. Introduction

Kidney transplantation is currently the standard treatment for patients with end-stage renal disease. Studies have shown that kidney transplantation allows for higher survival rates and better quality of life compared to dialysis patients. Therefore, kidney transplantation currently represents the gold standard in the treatment of end-stage renal disease [1,2,3]. To prevent transplant rejection and maintain allograft stable function, immunosuppressive therapy should be administered [4].

Immunosuppressive drugs can affect several types of cells, ultimately reducing inflammation and suppressing the body’s immune response. In kidney transplant recipients, immunosuppressive therapy is typically divided into two stages based on the duration of use: induction therapy and maintenance therapy [5,6]. Induction therapy involves biological agents administered at or around the time of transplantation. Two strategies are commonly used: the administration of antilymphocyte biological agents in combination with lower doses of conventional drugs, or the use of high doses of conventional immunosuppressive drugs [4,5]. Maintenance immunosuppression, on the other hand, begins at the time of transplantation and continues long term. This therapy most commonly uses immunosuppressive drugs in combination regimens. Current maintenance immunosuppression regimens include calcineurin inhibitors, mammalian target of rapamycin kinase inhibitors, antimetabolite agents, glucocorticoids, and biological immunosuppressive agents [3,6]. In some cases, a different classification of immunosuppressive drugs is used based on their use in the treatment of acute T cell-mediated (cellular) rejection and acute antibody-mediated (humoral) rejection [7].

The choice of immunosuppressive therapy depends on many clinical factors. The choice of a specific set of drugs is influenced primarily by the stage of treatment after transplantation (during the induction period or during the long-term maintenance of transplant function). The underlying cause of the disease leading to the need for organ transplantation and the current condition of the organ, including its degree of function and the risk of rejection, are also important. Immunosuppressive therapy should be tailored carefully to the needs of individual patients based on their general health, comorbidities, immune profile, and response to previous treatment [1,8].

Long-term immunosuppressive therapy with calcineurin inhibitors can lead to numerous adverse effects involving various organs and systems. In addition to having increased risks of cardiovascular disease [9] and nephrotoxic [10] and hepatotoxic [2] effects, these drugs can also cause metabolic and hormonal disorders. Furthermore, numerous clinical and experimental studies have demonstrated the adverse effects of these drugs on male reproductive organs [11,12,13,14]. Notably, many men of reproductive age report the need to regain fertility after kidney transplantation, which poses additional therapeutic challenges.

The aim of this review is to describe effects of calcineurin inhibitors on individual organs of the male reproductive system, hypothalamic–pituitary–gonadal (HPG) axis and semen parameters, as well as possible pathomechanism. This review provides a comprehensive synthesis of the direct and indirect reproductive effects of cyclosporine A (CsA) and tacrolimus (FK506) on different parts of the male reproductive system. This organ-by-organ analysis, which is the first to evaluate these organs together, covers the testis, epididymis, and accessory glands. While earlier reviews have largely summarised clinical observations, this work integrates experimental, clinical and in vitro evidence, providing a detailed analysis of molecular pathomechanisms including calcineurin signalling disruption, oxidative stress, mitochondrial dysfunction and fibrotic remodelling. This integrative approach enables clearer differentiation between drug-specific gonadotoxic effects and transplant-related confounding factors, providing a better understanding of male fertility outcomes in kidney transplant recipients.

## 2. Mechanism of Action of Calcineurin Inhibitors

Calcineurin inhibitors include CsA and FK506, which have different chemical structures but exhibit similar mechanisms of action. Both compounds form complexes with cytoplasmic proteins called immunophilins. The resulting complexes then interact with calcineurin, leading to its inhibition and, consequently, to the blocking of T-cell activation and the immune response [15].

Calcineurin, also known as protein phosphatase 2B (PP2B), is a Ca^2+^/calmodulin-dependent heterodimeric serine-threonine phosphatase composed of a catalytic subunit A (three isoforms were identified: protein phosphatase 3 catalytic subunit alpha (PPP3CA), protein phosphatase 3 catalytic subunit beta (PPP3CB), and protein phosphatase 3 catalytic subunit gamma (PPP3CC)) and a regulatory subunit B (two isoforms were identified: protein phosphatase 3 regulatory subunit B alpha (PPP3R1) and protein phosphatase 3 regulatory subunit B beta (PPP3R2)), which binds calcium ions via EF-hand motifs. In its resting state, the enzyme is inactive, and its activation occurs upon binding of the Ca^2+^/calmodulin complex to the regulatory domain and exposure of the catalytic center. Calcineurin acts as a calcium signal transducer by dephosphorylating a number of substrates. Among these, factors from the nuclear factor of activated T cells (NFAT) family are key for transcription [16,17,18]. Following dephosphorylation by calcineurin, NFAT translocates to the cell nucleus where it interacts with transcription partners, including activator protein 1, to initiate the expression of calcium-dependent genes. These include cytokine genes, receptors and effector proteins involved in cell differentiation [17,19].

CsA is a highly lipophilic cyclic peptide first isolated in 1971 from the filamentous fungus *Tolypocladium inflatum*. In 1983, CsA was introduced into clinical practice as a drug with immunomodulatory and immunosuppressive properties, enabling the immunological regulation of T lymphocytes [20,21]. The mechanisms of action of CsA involve its immunomodulatory effects through several pathways. CsA inhibits the activation and infiltration of T lymphocytes and blocks the release of specific inflammatory cytokines (Figure 1) [21].

After it penetrates T lymphocytes, CsA binds to cytosolic cyclophilins, primarily cyclophilin A, which belongs to the immunophilin family, with high affinity. The resulting drug-immunophilin complex binds to the surface of calcineurin in a groove that encompasses the A and B subunits. This blocks access to the catalytic centre for substrates and inactivates phosphatase activity, without affecting Ca^2+^/calmodulin binding [16,22]. The result is the inhibition of the dephosphorylation of nuclear regulatory proteins, including the NFAT, which is retained in the cytoplasm in a phosphorylated form [17]. As a result, these proteins are not translocated from the cytoplasm to the cell nucleus, where they regulate the expression of genes encoding numerous cytokines, including interleukin 2 (IL-2) and interleukin 4 (IL-4), which constitute the basic mechanism of immunosuppression used in the prevention of transplant rejection [22,23]. CsA also increases the expression of transforming growth factor β (TGFβ), which inhibits IL-2-stimulated T-cell proliferation and may exert profibrogenic effects [24].

Tacrolimus is a lipophilic macrolide antibiotic that was first obtained in 1984 from a fermentation culture of the soil bacterium *Streptomyces tsukubaensis* [25]. The immunosuppressive effect of FK506 was confirmed in 1987, and subsequent studies have shown that this drug can serve as an effective alternative to CsA, especially in the context of transplantology and the prevention of transplant rejection [24,26]. The mechanism of action of FK506 involves the inhibition of cellular immunity and selected elements of humoral immunity. Although FK506 has a similar mechanism of action to CsA, it has a lower molecular weight and a stronger effect [15,26]. FK506, like CsA, affects signal transduction in T lymphocytes, resulting in reduced expression of genes encoding key cytokines responsible for the activation and proliferation of these cells (Figure 1). Upon entering T lymphocytes, FK506 binds to proteins from the FK506-binding protein (FKBP) family, including FKBP12 and the FKBP12.6 isoform (FKBP1B), forming a highly specific complex. This complex then binds to calcineurin and blocks its enzymatic activity. As a result, calcium-dependent signaling pathways are inhibited, and transcription factors that regulate the transcription of cytokine-encoding genes are inactivated [24,26,27].

By inhibiting calcineurin activity, CsA and FK506 prevent the transcription of NFAT. As a result, these cells remain in a resting state (they do not progress from the G0 phase to the G1 phase of the cell cycle), and key cytokines such as IL-2, IL-4, and interferon γ (IFN-γ) are not synthesized. In addition, calcineurin inhibitors affect other signaling pathways associated with T-cell activation, including nuclear factor kappa-light-chain-enhancer of activated B cells (NF-κB), c-Jun N-terminal kinase and p38, the E twenty-six (ETS)-like transcription factor 1 (Elk-1), and nitric oxide synthase. Since both drugs inhibit IL-2 synthesis, their use may also inhibit the formation of T-reg lymphocytes, whose differentiation is dependent on this cytokine [15,21].

## 3. Influence of Calcineurin Inhibitors on Testis

### 3.1. Cyclosporine Monotherapy

Accumulating evidence from experimental and clinical studies demonstrates that CsA monotherapy exerts detrimental effects on the male gonads. The reported alterations involve both the seminiferous tubules and the testicular interstitium. These findings indicate that CsA adversely affects both the germinal and endocrine compartments of the testes [12,28,29,30,31,32,33,34,35].

#### 3.1.1. Experimental Studies

Numerous experimental studies have demonstrated that CsA induces a dose-dependent reduction in testicular weight [12,29,33]. Significant decreases have been reported following oral or subcutaneous administration of CsA at doses ranging from 10 to 40 mg/kg body weight [12,29,30,33,34,35]. However, other studies reported no significant reduction in testicular weight following treatment with 30 mg/kg CsA for 2 weeks or 10 mg/kg CsA for 21 or 120 days, suggesting that changes in testicular weight depend not only on the administered dose but also on the duration of exposure [30,32,36].

Several experimental studies have consistently confirmed that CsA induces dose- and treatment duration-dependent histopathological changes in the testes of rodents [12,29,30,31,33,34,36]. In rats, 14-day administration of CsA at 10 mg/kg induced only mild disruption of germ cell organization, whereas treatment with 20 or 40 mg/kg resulted in marked impairment of spermatogenesis. Higher doses resulted in degeneration of primary spermatocytes, spermatids, and Sertoli cells, vacuolization, and loss of Sertoli cell–germ cell connections. At 40 mg/kg, severe degeneration of the seminiferous tubules, with many containing only spermatogonia and Sertoli cells was revealed [29]. However, no significant changes in seminiferous tubule diameter were observed in neonatal rats following 120 days of treatment with 10 mg/kg CsA [30]. In turn, in kidney-transplanted rats receiving 10 mg/kg CsA for 24 weeks, testicular atrophy was characterized by reduced seminiferous tubule diameter, degeneration of the germinal epithelium and desquamation of the germ cells, loss of spermatids, and disorganization or absence of germ cell layers. Ultrastructural analysis additionally revealed a reduced number of mature spermatids and alterations in Sertoli cell organelles [34]. Similarly, treatment with 10 or 15 mg/kg CsA for 21 days resulted in decreased seminiferous tubule diameter and germinal epithelium thickness, accompanied by tubular atrophy, germ cell necrosis and degeneration, spermatogenic arrest, and exfoliation of immature germ cells into the tubular lumen [32,33]. Comparable lesions, including seminiferous tubule atrophy, impaired spermatid development, germ cell loss, and disruption of germ cell organization, were also reported following administration of 25–40 mg/kg CsA for 2–8 weeks [12,31,36]. Moreover, numerous residual bodies were observed following administration of 40 mg/kg CsA for 2 weeks, suggesting impaired phagocytic capacity of Sertoli cells. Additionally a detailed characterisation revealed the cytoplasmic profiles of spermatids at various stages of degeneration [31].

In addition to its effects on the seminiferous tubules, CsA has been shown to induce structural changes in the testicular interstitium of rodents. Treatment with CsA (10–40 mg/kg) for 2–24 weeks resulted in structural and functional alterations of the testicular interstitium, including fibrosis, capillary congestion, interstitial edema, and Leydig cell dysfunction [12,29,33,34,37]. Moreover, the other authors revealed that in rats, administration of 20 mg/kg CsA caused significant Leydig cell atrophy without altering cell number. They suggested that Leydig cell atrophy was mainly a consequence of decreased mitochondrial compartment and smooth endoplasmic reticulum content, decreased Golgi apparatus and peroxisome volumes, and a marked accumulation of lipid droplets. Moreover, these alterations were associated with impaired steroidogenesis and reduced testosterone production [37]. Ultrastructural alterations in the testicular interstitium were also observed at lower CsA doses. Specifically, in rats treated with 10 mg/kg body weight for 24 weeks resulted in dilation of the endoplasmic reticulum in Leydig cells [34].

#### 3.1.2. Clinical Studies

Compared with experimental studies, clinical evidence on the effects of CsA monotherapy on the human male gonad remains limited. This likely reflects the widespread use of combination immunosuppressive therapy in clinical practice, which limits the possibility of assessing the isolated effects of CsA. Nevertheless, the available data indicate that treatment with CsA at a dose of 8–10 mg/kg/day may significantly reduce testicular volume in men [28].

### 3.2. Tacrolimus Monotherapy

#### 3.2.1. Experimental Studies

Experimental studies indicate that FK506 monotherapy may affect the male gonads. However, the reported findings are inconsistent and appear to depend on treatment duration, dose, and the experimental model [12,36,38,39]. Available experimental evidence suggests that, under certain conditions, FK506 may produce less pronounced testicular histopathological alterations than CsA [12].

The effect of FK506 on testicular weight remains inconclusive. A significant reduction in testicular weight has been reported in rats treated with 1 mg/kg body weight for 60 days [38], whereas no significant changes were observed following administration of 1–3 mg/kg body weight for 2 weeks [36,38] or 0.8 mg/kg body weight for 8 weeks, even in animals subjected to unilateral nephrectomy [12]. Moreover, after 30 days of treatment with 1 mg/kg body weight, the reduction in testicular weight was not statistically significant, suggesting that the effects of FK506 on testicular weight depends primarily on the duration of treatment [39].

The effect of FK506 on testicular morphology remains inconsistent across studies and appears to depend on multiple experimental factors, including treatment duration, dose, and animal model, rather than on treatment duration alone. In short-term experiments, no abnormal changes were observed in the testis of rats receiving 1–3 mg/kg/day FK506 [36,38]. Similarly, in unilateral nephrectomized rats treated with FK506 (0.8 mg/kg body weight) for 8 weeks grossly normal seminiferous tubule architecture was exhibited, with only a slight reduction in sperm and spermatids and a mild, statistically insignificant increase in testicular injury score [12]. Administration of FK506 at a dose of 1 mg/kg body weight for 30 or 60 days resulted in irregularly shaped seminiferous tubules with disorganization of the spermatogenic epithelium, detachment of germ cells into the tubular lumen, and tubular atrophy associated with a significant reduction in tubular and epithelial areas [39]. These alterations were accompanied by a significant decrease in the number of spermatocytes and spermatids per tubule, although the reduction was more pronounced after 30 days than after 60 days of treatment. Sertoli cells exhibited irregularly shaped nuclei with densely stained chromatin, frequent perinuclear vacuolization, and abnormal displacement of their nuclei toward the adluminal compartment [39]. The more severe abnormalities compared with earlier studies may be explained by the substantially longer treatment period, as previous investigations [36,38] evaluating only 2 weeks of FK506 administration did not reveal significant histopathological alterations in the testes. Interestingly, not all studies indicate detrimental effects of FK506 on the testes. Martin et al. [40] demonstrated that FK506 exerted a protective effect against cadmium-induced testicular injury in rats, suggesting that under specific pathological conditions the drug may attenuate toxic damage to the male gonads.

In contrast to its effects on the seminiferous tubules, evidence regarding the influence of FK506 on the testicular interstitium remains limited [38,39]. Treatment with FK506 (1 or 2 mg/kg for 2 weeks) did not induce morphological alterations in Leydig cells of rats. Furthermore, in vitro experiments showed that FK506 did not affect Leydig cell viability or testosterone secretory capacity, indicating that short-term exposure does not impair Leydig cell function [38]. Similarly, Caneguim et al. [39] found no evidence of structural alterations in the testicular interstitium. The histopathological lesions were primarily localized to the seminiferous tubules, with only morphological irregularities of the peritubular tissue, manifested by irregular contours and occasional pronounced folding [39].

#### 3.2.2. Clinical Studies

No clinical studies evaluating the effects of FK506 monotherapy on the human male gonad were identified. This is most likely because FK506 is typically used in multidrug immunosuppressive regimens and is rarely administered as a single immunosuppressive agent in clinical practice.

### 3.3. Multidrug Protocols with Calcineurin Inhibitors

Multidrug immunosuppressive regimens constitute the standard approach to the prevention of allograft rejection after transplantation. Despite their widespread clinical use, data regarding their effects on testicular morphology are limited. No clinical studies evaluating histopathological changes in the testes of men receiving combination immunosuppressive therapy have been identified, and the available data are derived only from a small number of experimental animal studies [14,41].

#### 3.3.1. Experimental Studies

Experimental evidence regarding the effects of multidrug immunosuppressive regimens on testicular morphology remains scarce. The available studies [14,41] indicate that combination therapy may induce structural alterations in both the seminiferous tubules and the testicular interstitium, although the severity of these changes appears to depend on the immunosuppressive protocol. Grabowska et al. [14] demonstrated that daily oral administration of calcineurin inhibitor-based triple immunosuppressive regimens for six months, consisting of either CsA (5.0 mg/kg), MMF (20.0 mg/kg), and prednisone (4.0 mg/kg) or FK506 (4.0 mg/kg), MMF (20.0 mg/kg), and prednisone (4.0 mg/kg), induced histopathological alterations in the testes of male rats. However, after 6 months of treatment the reduction in testicular weight was not statistically significant. In turn, the observed changes included disorganization of the seminiferous epithelium, abnormal arrangement of germ cells, multinucleated germ cells, premature exfoliation of germ cells into the tubular lumen, intercellular vacuolization and a reduced number of germ cells. In addition, the authors reported significant reductions in both the diameter and the cross-sectional area of the seminiferous tubules [14]. Similar findings were reported by Freus et al. [40], who investigated the effects of prenatal exposure to calcineurin inhibitor-based triple immunosuppressive regimens. Female rats received CsA (2.5 mg/kg), MMF (10.0 mg/kg), and prednisone (4.0 mg/kg); FK506 (2.0 mg/kg), MMF (10.0 mg/kg), and prednisone (4.0 mg/kg); or CsA (2.5 mg/kg), EVL (0.25 mg/kg), and prednisone (4.0 mg/kg) for two weeks prior to pregnancy and during 3 weeks of pregnancy. Histological evaluation of the testes of sexually mature male offspring demonstrated reduced testicular weight, disorganization of the seminiferous epithelium, depletion of germ cells, premature detachment of immature germ cells into the tubular lumen, and vacuolization of both the seminiferous epithelium and Sertoli cells [41].

Evidence regarding the effects of calcineurin inhibitor-based multidrug immunosuppressive regimens on the testicular interstitium remains limited. In a six-month experimental study, Grabowska et al. [14] demonstrated a mild accumulation of collagen fibres in the perivascular regions of the testes, shrinkage of the interstitial tissue, and loss of structural connections between the seminiferous tubules and the surrounding interstitium. No increased collagen deposition was detected in the lamina propria of the seminiferous tubules, and its thickness remained unchanged. Moreover, no visible structural alterations were observed in the tunica albuginea [14]. Changes in the interstitial compartment were also observed in the testes of adult male offspring exposed in utero to the combination of CsA, MMF, and prednisone. Freus et al. [41] described abnormally organized, hypertrophic interstitial tissue containing numerous eosinophilic cells, particularly in the vicinity of invaginated seminiferous tubules.

#### 3.3.2. Clinical Studies

The clinical trials did not include a histopathological evaluation of the testes. In a few cases, only an ultrasound evaluation of testicular volume was performed [11,42]. In a group of eight patients receiving a calcineurin inhibitor (CsA or FK506) in combination with a purine synthesis inhibitor (azathioprine (AZA) or mycophenolate mofetil (MMF)) and prednisone, normal testicular volume was observed in all participants after a follow-up period of 41.5–90.5 months [42]. In contrast, among men with a normally functioning transplant who were at least 15 months post-transplant and receiving stable maintenance therapy with prednisone in combination with a calcineurin inhibitor, whose dosage was adjusted based on trough concentrations, testicular volume was smaller than in the control group. Furthermore, in patients with testosterone levels below the reference range, testicular volume was significantly lower than in patients with normal testosterone levels [11].

## 4. Influence of Calcineurin Inhibitors on Epididymis

### 4.1. Cyclosporine A Monotherapy

Compared with the testes, considerably less is known about the effects of CsA on the epididymis. No comprehensive clinical studies evaluating epididymal morphology in men receiving CsA have been identified, and the available data are based almost entirely on a limited number of experimental animal studies. These studies suggest that CsA induces relatively mild structural alterations in the epididymis but may substantially impair epididymal sperm maturation and quality [29,31,33].

#### Experimental Studies

The obtained experimental results concerning the effects of CsA on the epididymis remain inconsistent. Seethalakshmi et al. demonstrated a dose-dependent decrease (doses ranging from 10 to 40 mg/kg body weight for 14 days) of epididymal weight [29], whereas treatment with 10, 15 or 30 mg/kg body weight for 14 or 21 days resulted only in a slight, statistically insignificant decrease [32,33,36]. In turn, in rats administered CsA at a dose of 15 mg/kg body weight for 56 days epididymal weight was significantly reduced [43]. These discrepancies suggest that the effect of CsA on epididymal weight depends on the dose and time of exposure.

There are limited experimental data describing detailed morphological changes in the epididymis during CsA therapy. Hisatomi et al. [36] have found eosinophilic globules in the duct of the epididymis after administration of CsA at a dose of 30 mg/kg body weight for 2 weeks. The abnormalities reported in the literature primarily involve the effects of CsA on the cellular components of the epididymal duct, particularly spermatozoa [29,32,33]. Ultrastructural examination after treatment with 40 mg/kg CsA for 2 weeks revealed in the lumen of the epididymal duct of rats, cellular components, identifying sperm conglomerates (in some cases incorrectly identified by authors as residual bodies) containing abnormal sperm cells and their fragments (unusual cellular configurations with two heads and flagella, discontinuities between sperm heads and flagella, widened subacrosomal space, significant abnormalities in the axoneme, outer dense fibres, and mitochondrial sheath). These findings suggested that CsA may impair the structural integrity and maturation of testicular and epididymal sperm [31].

### 4.2. Tacrolimus Monotherapy

#### Experimental Studies

As with CsA, evidence regarding the effects of FK506 on the epididymis remains limited. The available experimental studies suggest that FK506 may affect epididymal weight and function [36,44].

The reported findings are inconsistent. Some authors demonstrated a significant dose-dependent reduction in epididymal weight following subcutaneous administration of FK506 at doses of 2 or 3 mg/kg body weight for 2 weeks [44], whereas others observed no statistically significant changes in rats treated with 1 or 3 mg/kg body weight over the same treatment period [36].

Histopathological examination has shown only eosinophilic globules in the duct of the epididymis after administration of FK506 at a dose of 3 mg/kg body weight [35]. Other research revealed a dose-dependent decrease in L-carnitine, a key component of epididymal secretions that plays important roles in sperm maturation and motility. However, no changes were noted in α-glucosidase, acid phosphatase, and antioxidant enzyme activities [44].

### 4.3. Multidrug Protocols with Calcineurin Inhibitors

There are few data directly describing the effects of multidrug regimens on the epididymis. The available experimental and clinical studies suggest that combination therapy may induce mild morphological alterations in the epididymis, although the evidence is limited [45,46].

#### 4.3.1. Experimental Studies

Previous preliminary studies by our group demonstrated that six months of daily oral administration of calcineurin inhibitor-based triple immunosuppressive regimens, consisting of either CsA (5.0 mg/kg), MMF (20.0 mg/kg), and prednisone (4.0 mg/kg) or FK506 (4.0 mg/kg), MMF (20.0 mg/kg), and prednisone (4.0 mg/kg), induced morphological changes in the epididymis of male rats. The observed alterations included collapse of the epididymal duct wall and a reduction in the height of the epididymal epithelium [45].

#### 4.3.2. Clinical Studies

In the only available clinical study, Bozzini et al. [46] reported the presence of epididymal microcalcifications in a selected group of young renal transplant recipients receiving calcineurin inhibitors in combination with MMF. However, due to their history of ESRD and previous dialysis, it is not possible to establish a causal relationship with calcineurin inhibitor-based immunosuppression [46].

## 5. Influence of Calcineurin Inhibitors on Accessory Glands

### 5.1. Cyclosporine A Monotherapy

#### Experimental Studies

In contrast to the testis and epididymis, the prostate has been comparatively poorly characterized as a target of CsA, and the available evidence is derived almost exclusively from rodent models [43]. No histopathological or ultrastructural study has evaluated prostatic tissue in men receiving CsA.

At the organometric level, studies conducted in rats have demonstrated that CsA administration at doses ranging from 10 to 40 mg/kg body weight for 2–8 weeks results in a significant, dose-dependent reduction in prostate weight [29,33,36,43]. In contrast, findings regarding the seminal vesicles remain inconsistent. Significant decreases in seminal vesicle weight have been reported following treatment with 10–40 mg/kg CsA for 14 days [29,36], whereas no significant changes were observed after administration of 15 mg/kg CsA for 21 or 56 days [33,43]. Furthermore, treatment with 15 mg/kg CsA for 56 days significantly reduced the weight of the coagulating glands [43].

In contrast to the organometric changes, the morphological and functional consequences of CsA monotherapy exposure on the prostate have been investigated only to a limited extent. Freitas et al. demonstrated that CsA at a dose of 15 mg/kg body weight for 56 days in rats led to focal atrophy of the ventral prostate glandular epithelium and a reduction in the absolute volume of the luminal, epithelial, and stromal compartments. These changes were accompanied by significant impairment of the secretory function of epithelial cells, manifested by a regression of the rough endoplasmic reticulum and Golgi apparatus. These findings indicate impaired prostatic secretory activity, which may alter the composition of prostatic fluid and thereby adversely affect sperm viability [43].

### 5.2. Tacrolimus Monotherapy

#### Experimental Studies

Evidence directly addressing the influence of FK506 on the prostate and the remaining accessory sex glands is even more limited than that available for CsA, and it is confined to organometric data obtained in single rodent models [36,44]. No morphological or functional study has characterized prostatic tissue under FK506 exposure in men.

In rats, subcutaneous administration of FK506 at 1, 2, and 3 mg/kg body weight for two weeks caused a significant, dose-dependent reduction in the weights of both the prostate and the seminal vesicles [36,44]. However, no histopathological alterations were detected, leaving the functional significance of the reduced organ weights unclear [36].

### 5.3. Multidrug Regimens with Calcineurin Inhibitors

Data regarding the effects of calcineurin inhibitor-based multidrug immunosuppressive regimens on the prostate are limited to small experimental studies [13,47,48], and no human studies have directly evaluated prostate morphology during chronic immunosuppressive therapy.

#### 5.3.1. Experimental Studies

Experimental studies from our group showed that calcineurin inhibitor-based treatment with different combinations of immunosuppressive agents induced protocol-dependent morphological and ultrastructural alterations in the rat prostate [13,47,48]. Grabowska et al. [13,47,48] found that administration of CsA (5 mg/kg body weight) in combination with prednisone (4 mg/kg body weight) and sirolimus (0.5 mg/kg body weight) resulted in marked atrophic changes in the glandular epithelium, including a reduction in secretory organelles and lysosomal vesicles, together with the accumulation of numerous intracytoplasmic vacuoles [13,47,48]. In contrast, regimens combining a calcineurin inhibitor (CsA, 5 mg/kg body weight, or FK506, 4 mg/kg body weight) with MMF (20 mg/kg body weight) and prednisone (4 mg/kg body weight) were associated with the development of atypical focal hyperplasia of the glandular epithelium. These lesions were characterized by enhanced proliferation of luminal epithelial cells forming papillary projections into the glandular lumen, increased nuclear-to-cytoplasmic ratio, nuclear hyperchromasia, and focal epithelial stratification with multilayered nuclear arrangement [47,48]. Moreover, ultrastructural examination further demonstrated thickening of the nuclear envelope, prominent nucleoli, and dilation of the rough endoplasmic reticulum cisternae and Golgi apparatus. Moreover, these changes were accompanied by a significant increase in the number of CD117-positive mast cells within the prostatic tissue, suggesting activation of local inflammatory or tissue-remodelling processes [13].

#### 5.3.2. Clinical Studies

In humans, available evidence regarding the prostate during calcineurin inhibitor-based immunosuppressive therapy is confined almost exclusively to oncological outcomes rather than direct histopathological assessment [49,50]. Studies of solid-organ transplant recipients receiving long-term immunosuppressive therapy, predominantly based on calcineurin inhibitors, have not demonstrated an increased incidence of prostate cancer. In several cohorts, the standardized incidence ratio was even lower than that observed in the general population, a finding more likely attributable to differences in screening practices, recipient selection, and hormonal factors than to a protective effect of immunosuppressive therapy. Mechanistic evidence linking calcineurin inhibitors to prostatic carcinogenesis remains limited and inconclusive. Although dysregulation of the calcineurin/NFAT signalling pathway has been implicated in epithelial tumour biology, its specific contribution to prostate pathology during calcineurin inhibitor therapy has not been established [49,50].

## 6. Influence of Calcineurin Inhibitors on Hypothalamic–Pituitary–Gonadal Axis

### 6.1. Cyclosporine A Monotherapy

Experimental data indicate that CsA monotherapy may disrupt the function of the HPG axis; however, the direction and severity of hormonal changes depend on the dose, duration of exposure, animal model, and concomitant renal damage. The most commonly observed effect is a decrease in testosterone concentration, which in most studies is accompanied by an increase in gonadotropin concentration, which may indicate impaired testicular steroidogenesis and the development of compensatory hypergonadotropinemia [12,29,34,36,37,43].

#### 6.1.1. Experimental Studies

In an experimental model, rats receiving CsA at doses of 10, 20, or 40 mg/kg body weight showed a dose-dependent reduction in serum testosterone concentrations. At a dose of 40 mg/kg body weight, testosterone concentrations were even below the detection limit of the assay used. Simultaneously, a significant 3- to 6-fold increase in luteinising hormone (LH) concentration and a significant increase in follicle-stimulating hormone (FSH) concentration were observed in all CsA-treated groups [29]. This hormonal profile suggests primary impairment of testicular function, involving Leydig cells and spermatogenesis, with a preserved pituitary response to reduced androgen negative feedback.

A similar decrease in plasma testosterone concentration was demonstrated in rats after administration of CsA at a dose of 15 mg/kg body weight for 56 days [43]. Monthly exposure to CsA at a dose of 20 mg/kg body weight also significantly reduced basal testosterone concentration. Furthermore, the testosterone response to human chorionic gonadotropin (hCG) stimulation was attenuated. While hCG caused an approximately four-fold increase in testosterone concentration in control animals, in CsA-treated rats, this increase was only two-fold. This result indicates a limited steroidogenic reserve of Leydig cells and suggests a direct peripheral effect of CsA on the testes. This functional deficit is fully congruent with the ultrastructural findings obtained in the same model, namely Leydig cell atrophy [37]. In a rat model following unilateral nephrectomy, administration of CsA at a dose of 25 mg/kg body weight for 8 weeks led to a significant reduction in testosterone concentration and an increase in estradiol concentration. Simultaneously, LH, FSH, and prolactin concentrations were elevated [12]. It is worth noting that increased estradiol may additionally modulate gonadotropin secretion and exacerbate androgen deprivation. It should be emphasized, however, that the unilateral nephrectomy model may independently influence the hormonal environment, so not all changes can be clearly attributed solely to CsA.

Not all studies confirm clear hormonal disturbances after CsA administration. After 2 weeks of subcutaneous administration of CsA at a dose of 30 mg/kg/day, no significant differences in testosterone or LH concentrations were found, although a slight increase in FSH was noted [36]. Similarly, no significant changes in testosterone, LH, or FSH concentrations were observed in rats receiving CsA at a dose of 10 mg/kg from day 11 to week 24 of life. Notably, the study documented pronounced testicular atrophy and degeneration of the germinal epithelium despite hormone levels remaining unchanged, indicating that structural damage to the seminiferous tubules may occur before measurable endocrine impairment [34].

#### 6.1.2. Clinical Studies

Clinical data on the effects of CsA monotherapy are limited because in humans, this drug is most often used in multidrug immunosuppressive regimens. Increased LH levels were noted in men after kidney transplantation who were receiving CsA at a dose of 8–10 mg/kg/day and had suboptimal graft function. The authors of the study concluded that the primary determinant of gonadal function was the function of the transplanted kidney, not the type of immunosuppressive treatment itself [28].

Consequently, when interpreting clinical findings, consideration of confounding variables is required in order to understand the clinical significance of CsA. Patients with chronic kidney disease who undergo prolonged dialysis are predisposed to uremic hypogonadism. Furthermore, glucocorticoid therapy and altered renal function independently modulate the hypothalamic–pituitary–gonadal axis, in addition to calcineurin inhibitor exposure. Distinguishing these transplant-related factors from direct calcineurin inhibitor gonadotoxicity is important in order to accurately assess the reproductive effects of specific drugs.

### 6.2. Tacrolimus Monotherapy

Available data suggest that the effects of FK506 on the HPG axis are generally weaker than those of CsA, especially during short-term treatment. However, experimental results are inconsistent and depend on the dose used, exposure time, and the presence of additional renal burden [12,36,38].

#### Experimental Studies

Experimental studies indicate that short-term administration of FK506 does not cause persistent impairment of basal testicular steroidogenic function or significant impairment of LH secretion. Rats receiving FK506 subcutaneously at doses of 1 or 3 mg/kg/day for 2 weeks showed no significant changes in testosterone or LH concentrations. In the 1 mg/kg/day group, only a slight increase in FSH concentrations was observed [36]. Similarly, in young adult rats receiving FK506 intramuscularly at doses of 1 or 2 mg/kg/day for 14 days, no significant reduction in basal testosterone concentrations was observed. LH concentrations remained within a range comparable to those observed in control animals. Furthermore, treatment with a dose of 2 mg/kg/day did not alter the testosterone response to hCG stimulation. At a dose of 1 mg/kg/day, a transiently lower testosterone concentration was observed 24 h after hCG administration, but the response to stimulation normalized 4 weeks after the end of therapy [38].

More prolonged exposure or concomitant renal damage may reveal mild impairment of testicular androgenic function, with secondary increases in LH and FSH. In a model of unilaterally nephrectomized rats administered FK506 at a dose of 0.8 mg/kg body weight for 8 weeks, a decrease in testosterone concentration was observed (although this decrease was less pronounced than in the group receiving CsA). Estradiol concentrations remained unchanged. Simultaneously, significant increases in LH, FSH, and prolactin concentrations were noted [12]. The increase in gonadotropins with decreased testosterone may indicate partial impairment of testicular hormonal function and compensatory activation of the pituitary axis.

Compared with CsA, FK506 appears to induce less severe impairment of steroidogenesis, but the number of available studies is limited [12]. No clinical trials assessing the effect of FK506 monotherapy on the HPG axis in men have been identified.

### 6.3. Multidrug Regimens with Calcineurin Inhibitors

In clinical practice, calcineurin inhibitors are primarily used in combination with glucocorticosteroids and antiproliferative drugs such as AZA or MMF. Therefore, assessing the effect of calcineurin inhibitor monotherapy on the HPG axis is difficult. The observed hormonal disturbances may result from the calcineurin inhibitor activity, as well as from chronic kidney disease, dialysis, graft function, prednisone use, and concomitant metabolic disorders [11,51,52,53,54,55,56,57,58,59,60,61].

#### Clinical Studies

When interpreting the influence of multidrug immunosuppressive regimens, it is important to distinguish calcineurin inhibitor-specific effects from the cumulative impact of chronic kidney disease severity, the duration and intensity of dialysis exposure, glucocorticoid dosage, and residual graft dysfunction. These factors independently suppress gonadotropin and testosterone production, and must be considered when evaluating the relative contribution of calcineurin inhibition to the observed hormonal abnormalities.

Clinical studies have demonstrated a transient deterioration of the HPG axis in the early post-transplant period, followed by progressive recovery [51,52,53]. Prior to surgery, reduced levels of testosterone, free testosterone, FSH and LH were found in men before kidney transplantation. One month after transplantation, during treatment with CsA, AZA, and methylprednisolone, significant decreases in testosterone, free testosterone, androstenedione, dehydroepiandrosterone sulfate (DHEAS), sex hormone-binding globulin (SHBG), estradiol, and prolactin were observed, whereas gonadotropin levels (LH and FSH) remained stable. Free testosterone returned to pre-surgical levels after three months, total testosterone after six months, and all measured hormones normalized within twelve months. Importantly, the CsA dose remained constant throughout the study, while AZA and methylprednisolone were progressively reduced from the second postoperative day. This indicates that recovery of the HPG axis occurred alongside steroid reduction rather than due to a change in calcineurin inhibitors exposure [51]. A gradual restoration of serum prolactin, LH and testosterone was observed in male recipients treated with CsA, AZA and prednisone, with a similar time course. There was no significant difference from controls 3–4 months after transplantation [52]. The same pattern was documented in cases outside of kidney transplantation [53]. In men after heart transplantation who were receiving CsA and prednisone, the lowest free and total testosterone concentrations occurred during the first month, when the prednisone dose was highest. These concentrations then normalised by the second month. Meanwhile, LH, FSH, estradiol and SHBG were at their lowest in the first month, increasing between months two and six. Importantly, low testosterone levels were associated with prednisone dosage, but not with CsA dosage [53]. The absence of a compensatory gonadotropin rise in the face of low testosterone in the early post-transplant period indicates functional, centrally mediated hypogonadism that is most plausibly driven by glucocorticoids and stress rather than being specific to calcineurin inhibitors.

Cross-sectional studies performed on stable recipients months to years after transplantation have generally reported normal hormonal profiles [42,54,55,56,57]. For example, eight out of nine men receiving CsA (1.6–5 mg/kg/day) and AZA had normal testosterone levels, and most of these men had FSH and LH levels within the reference range. Furthermore, three out of four couples attempting conception became pregnant [54]. Normal testosterone and FSH levels were also reported in four recipients treated with CsA (4 mg/kg/day) and prednisolone (0.15 mg/kg/day), despite two of them being infertile. These observations demonstrated that a normal endocrine profile does not exclude impaired fertility, suggesting that the reproductive defect in these patients may reside at the level of spermatogenesis and sperm quality rather than the HPG axis [55]. In a larger cohort of 55 male recipients treated with CsA (3 mg/kg/day), prednisone (10 mg/day) and AZA (75 mg/day) for 12–120 months, no significant differences in FSH, prolactin or testosterone were detected [56]. Similar results were reported by other authors [41,56]. In a small cohort, treated with a calcineurin inhibitor (CsA or FK506) combined with a purine synthesis inhibitor (AZA or MMF), as well as prednisone, normal LH, FSH and testosterone levels after a mean follow-up period of 41.5–90.5 months were noted [42]. Normalisation of LH and FSH levels was also observed in patients receiving CsA, AZA and prednisone [57]. Other studies have compared men treated with either CsA or FK506 and low-dose methylprednisolone. The authors found that FSH, LH, prolactin and total testosterone were within the normal range, with no statistical difference between the two calcineurin inhibitors [58]. Nevertheless, this comparison is limited by the small sample size and the lack of information on drug doses or treatment duration. Interestingly, in 40 recipients treated with CsA (1.3–2.0 mg/kg/day), prednisone (5–10 mg/day) and AZA or MMF, prolactin, LH and FSH levels were significantly lower, while testosterone levels were significantly higher, compared to those of uremic patients [59]. This suggests that successful transplantation may reverse the hypogonadism and hyperprolactinaemia associated with end-stage renal disease, at least partially. These results contrast with those of a study of 20 men with fully functioning allografts at least 15 months after transplantation, who were receiving stable maintenance therapy comprising prednisone (reduced to 8 mg daily) combined with trough-level-guided calcineurin inhibitor (CsA or FK506) in most cases, and MMF or AZA in a subgroup. Testosterone, DHEAS and androstenedione levels were significantly lower, while 17-hydroxyprogesterone levels were significantly higher, compared to controls. Prolactin, estradiol and gonadotropin levels did not differ between the groups. Testosterone levels were below the normal range in 14 patients and within the lowest tertile in six patients. However, LH levels were only mildly increased in three patients and FSH levels exceeded the normal range in four patients, suggesting concomitant damage to spermatogenesis. No significant difference in gonadotropin levels was found between patients with low and low-normal testosterone levels. These endocrine abnormalities were accompanied by organometric changes and a high prevalence of clinical symptoms. The failure of LH to rise appropriately in the presence of overt testosterone deficiency suggests a mixed primary and central defect. Meanwhile, the concurrent reduction in adrenal androgens is most consistent with chronic corticosteroid-induced suppression of adrenocorticotrophin hormone (ACTH) secretion [11].

In recipients treated with AZA, prednisone, and CsA reduced testosterone levels were observed alongside normal FSH and LH levels, as well as an absent gonadotropin response to gonadotrophin-releasing hormone (GnRH) stimulation. The authors of this study concluded that CsA acts both centrally, by inhibiting gonadotropin secretion, and peripherally, by reducing testicular testosterone production [60]. Approximately 16 ± 2 months after kidney transplantation, partially consistent findings were found in prospectively analysed hormonal profiles. Lower FSH and estradiol levels were observed in patients receiving CsA and prednisone, independently of allograft function. No significant differences were found in regimens combining CsA, AZA and prednisone. In that study, the CsA dose was found to correlate inversely with estradiol and directly with total testosterone, while the duration of pre-transplant dialysis was found to correlate with both FSH and LH [61]. This confirms the significant influence of the pre-transplantation state on gonadal axis function.

Overall, the available clinical data suggest that multidrug immunosuppressive regimens based on calcineurin inhibitors may be associated with transient hypogonadism, which is most often of a hypogonadotropic nature and occurs in the first weeks or months after transplantation. In most patients, the function of the HPG axis gradually normalises as transplant function improves and glucocorticoid doses are reduced. However, some long-term recipients experience persistently low testosterone levels alongside inadequately normal gonadotropin concentrations. Studies suggest that exposure to glucocorticosteroids and transplanted organ function, rather than calcineurin inhibitor use, are the primary determinants of these disorders [51,53]. To date, no clear differences have been demonstrated in the effects of CsA- and FK506-based regimens on HPG axis function [58]. However, the interpretation of the available results is significantly limited by the poor methodological quality of the selected studies. Most of the studies included small patient groups and were cross-sectional. Furthermore, drug doses and the duration of therapy were often not reported [42,52,58,61]. Pre-transplant hormonal results were also typically missing, and analyses rarely considered potential confounding factors such as graft function, cumulative glucocorticoid exposure, duration of dialysis, age and comorbidities. Dynamic assessment of the hormonal axis using a GnRH or hCG stimulation test has only been performed sporadically [60].

## 7. Influence of Calcineurin Inhibitors on Semen Parameters

### 7.1. Cyclosporine A Monotherapy

Experimental studies provide evidence that CsA monotherapy impairs multiple seminological parameters in a dose- and treatment duration-dependent manner. The reported alterations included changes to sperm count, motility, morphology, and ultrastructural integrity, which have implications for male fertility [12,29,33].

#### 7.1.1. Experimental Studies

In rodents sperm concentration and motility were consistently reduced following CsA exposure [12,29,33]. In rats treated with 10, 20 and 40 mg/kg of body weight, a dose-dependent decrease in the number and motility of epididymal sperm was demonstrated. Significant reductions were observed in all groups. This decrease was proportional to the administered dose [29]. Similarly, subcutaneous administration of 15 mg/kg of CsA for 21 days significantly reduced epididymal sperm concentration and motility [33]. In unilaterally nephrectomised rats treated with 25 mg/kg body weight for eight weeks, a marked reduction in the total number of testicular spermatozoa and the number of motile spermatozoa, and increased sperm abnormalities were observed, indicating profound impairment of spermatogenesis [12]. Notably, even at the lowest examined dose (10 mg/kg body weight), sperm concentration and motility were significantly diminished yet fertility was only marginally affected, with 67% of matings resulting in pregnancy. Complete reproductive failure occurred at 20 and 40 mg/kg body weight [29].

Sperm morphology may be a sensitive indicator of CsA toxicity, with the observed alterations being dose-dependent. No sperm morphological abnormalities were evident at 10 mg/kg body weight for 14 days [29]. By contrast, epididymal spermatozoa from rats treated with 20 mg/kg exhibited distinctive morphological alterations, notably the presence of large vesicular cytoplasmic droplets retained in the midpiece, a feature absent in the control group. At the highest dose of 40 mg/kg, severe morphological damage was evident, with spermatozoa displaying fractured heads, missing tail end pieces and wrinkled head surfaces [29]. Similarly, 15 mg/kg of CsA for 21 days significantly increased the percentage of epididymal sperm abnormalities affecting the head, tail, and total sperm [33].

Particular emphasis should be placed on the retention of cytoplasmic droplets in the sperm midpiece, which is a hallmark of impaired epididymal sperm maturation, which involves, among other processes, the progressive shedding the cytoplasmic droplet, as well as structural, functional, and molecular changes in sperm cells [62]. Seethalakshmi et al. [29] demonstrated that CsA treatment impairs the migration of the cytoplasmic droplet to the distal end of the midpiece. This results in the retention of large vesicular droplets in the midpiece of a substantial proportion of spermatozoa, which is a common finding. This abnormality has been linked to sterility in rats, suggesting that CsA-induced epididymal dysfunction could be a cause of reduced fertility [29].

#### 7.1.2. In Vitro Studies

The performed studies revealed that CsA exerts direct toxic effects on human spermatozoa in vitro in a dose- and time-dependent manner. After 10 min of incubation, CsA at a concentration of 1 mg/mL resulted in near-complete loss of sperm motility. In contrast, a concentration of 200 µg/mL had no significant impact on sperm motility or viability over 60 min. Removing bovine serum albumin from the incubation medium increased sperm susceptibility to the drug. An increase in lactate dehydrogenase concentration, a marker of membrane damage, impaired sperm membrane integrity and increased membrane permeability when exposed to CsA [63].

### 7.2. Tacrolimus Monotherapy

Studies on the effect of FK506 on semen parameters are limited. Experimental data suggest that FK506 has a dose- and time-dependent effect on sperm parameters, which are generally less severe than those induced by CsA [12,36].

#### Experimental Studies

Short-term administration of FK506 at a dose of either 1–3 mg/kg/day for two weeks was found to significantly reduce sperm counts in the cauda epididymis. Impairment of motility was also documented. Notably, however, these changes in concentration and motility were generally less severe than those induced by CsA when administered at comparable doses for the same duration [36,44].

In a longer-term experiment involving a compromised renal model, rats that had undergone unilateral nephrectomy were administered FK506 at a dose of 0.8 mg/kg body weight for eight weeks. Unlike the results obtained with CsA in the same animal model, treatment with FK506 did not affect testicular sperm motility or the frequency of sperm abnormalities. The gonadal toxicity of FK506 in this model was substantially lower than that of CsA [12].

### 7.3. Multidrug Regimens with Calcineurin Inhibitors

There is relatively abundant clinical data on seminological parameters in recipients receiving multidrug immunosuppressive regimens, although this data are heterogeneous in design. Available studies indicate that the effect of multidrug immunosuppressive regimens on semen parameters depends mainly on the primary drug used. Collectively, the available clinical evidence suggests that, while acute post-transplant impairment of semen quality occurs in a substantial proportion of recipients, most men recover to near-normal or normal parameters within 1–2 years. Furthermore, the magnitude of any residual impairment appears to be generally modest and dose-related.

#### Clinical Studies

The clearest evidence for the reversibility of calcineurin inhibitor-induced sperm dysfunction comes from longitudinal studies documenting recovery of sperm quality after transplantation [52,56]. A significant initial impairment of sexual function was reported in 55 male kidney transplant recipients who were treated with CsA (3 mg/kg/day), prednisone (10 mg/day) and AZA (75 mg/day) for between 12 and 120 months. However, one year post-transplant, 72% of the cohort had resumed an active sex life. During follow-up, the 55 recipients fathered 30 pregnancies. At the time of assessment, 47.3% of patients exhibited normozoospermia, 18.2% asthenozoospermia and 14.5% oligozoospermia. The remainder (20%) displayed combinations of impaired count, motility and/or morphology. With respect to morphology, 88% of patients had 30% or more spermatozoa with a normal appearance. Regarding sperm motility, 65.5% of patients exhibited normal motility according to World Health Organization (WHO) criteria. Therefore, most of the assessed parameters were within normal limits for this population of men post-transplantation, indicating substantial recovery from the acute post-transplant deterioration [56]. A similar time course was reported during the evaluation of 78 married male recipients who were treated with CsA, AZA, and prednisone from the first post-operative day. Four months after transplantation, sperm concentration, motility, viability and the proportion of morphologically normal spermatozoa were all significantly higher than pre-transplant values, approaching those of a control group. This improvement highlights the temporal relationship between the restoration of the HPG axis and the recovery of sperm function [52].

In another study 212 male renal transplant recipients were receiving various combinations of CsA or FK506-based immunosuppressive therapy (predominantly calcineurin inhibitors with AZA or MMF, together with prednisone), after a mean exposure to CsA of 43.79 ± 12.48 months and a mean post-transplantation period of 46.91 ± 26.19 months. Notably, post-transplant conception in 212 recipients, resulting in 216 live-born children, was reported. Semen analyses from 19 recipients performed two years after transplantation showed no significant differences in sperm motility, motility rate, concentration, or normal morphology rate compared with a healthy control group [64]. The results suggest that spermatozoal parameters normalise in the long term in recipients who are maintained on therapeutic-range calcineurin inhibitors doses and have stable graft function.

Cross-sectional studies evaluating stable recipients months to years post-transplant have generally reported near-normal or normal sperm parameters [42,54]. A semen analysis was performed on nine men who were receiving CsA (at a dose of 1.6–5 mg/kg/day) and AZA. One patient exhibited azoospermia, while the others demonstrated normal motility and sperm concentration. Of the nine men, four engaged in unprotected intercourse and three partners conceived [54]. In another study, eight recipients received a calcineurin inhibitor in combination with a purine synthesis inhibitor (AZA or MMF) and prednisone, after mean follow-up periods of 41.5 and 90.5 months. One patient exhibited azoospermia, and six out of eight had one or more sperm abnormalities (oligozoospermia, asthenozoospermia or teratozoospermia in various combinations in four patients and oligoasthenoteratozoospermia in two patients) [42].

Cross-sectional comparative studies demonstrated modest differences between calcineurin inhibitors regimens [65]. Cao et al. compared 20 patients receiving FK506, MMF and prednisone with 17 recipients taking CsA, AZA and prednisone, as well as 15 healthy controls. No significant differences were found among the three groups in terms of sperm viability rate, curve line velocity or velocity of average path. However, the recipients treated with FK506 exhibited a lower rate of morphological anomalies, a higher percentage of progressive motility, and a higher straight-line velocity compared with the CsA group [65]. The authors concluded that combining FK506 with MMF could improve sperm mobility and morphology in kidney transplant recipients. This suggests that, in this comparison cohort at least, FK506-based regimens may be more effective than CsA-based protocols for improving spermatozoal outcomes.

The dose-dependent effects of CsA on sperm quality have been documented in patients receiving multidrug regimens [66]. The relationship between the dosage of the immunosuppressive agent and sperm parameters was also evaluated in kidney transplant recipients. It was found that sperm motility and the proportion of normal sperm morphology were significantly lower when the CsA dosage was within the range of 4.1–6 mg/kg/day compared to the control group. However, when the CsA dose was 2.1–4 mg/kg/day, there was an improvement in sperm motility and morphology. This indicates that CsA retains slight effects on sperm parameters even at this intermediate dose. Importantly, no significant effects on sperm parameters were observed when the CsA dose was in the range of 1.3–2 mg/kg/day. This suggests that the adverse effects of CsA on sperm quality may be reversible at low therapeutic doses. In the same group of recipients who were taking CsA in doses ranging from 1.2 to 3.0 mg/kg/day, 216 children were born following transplantation. Physical examinations revealed only minor incidental abnormalities, and all the children were found to be healthy [66]. This outcome suggests that low-dose calcineurin inhibitor multi-drug therapy is safe with respect to offspring.

Studies examining the relationship between CsA trough levels and sperm parameters provide additional insight [55]. Five groups of kidney transplant recipients were examined: two groups of infertile recipients and two groups of fertile recipients taking 4 mg/kg/day of CsA and prednisolone; five groups of infertile recipients and six groups of fertile recipients taking 1–3 mg/kg/day of AZA and prednisolone; and 11 groups of infertile recipients and eight groups of fertile recipients taking 4 mg/kg/day of CsA, 1 mg/kg/day of AZA and prednisolone. Among recipients taking CsA, significant decreases in sperm concentration, the percentage of motile spermatozoa, straight-line velocity (VSL), linearity and velocity of the average path were evident in the infertile group compared with the fertile group. Notably, there was an inverse correlation between sperm concentration and VSL and CsA whole-blood trough levels [55]. Higher systemic CsA exposure was associated with poorer sperm parameters and supported the rationale for maintaining calcineurin inhibitors levels within the lower therapeutic range, particularly for recipients who were concerned about fertility.

Another study compared the sperm parameters of 40 renal transplant recipients who were treated with CsA (1.3–2.0 mg/kg/day), prednisone (5–10 mg/day) and AZA (50 mg/day), or MMF (1.0 g/day), with those of patients with uremia. The sperm motility, viability and concentration parameters, as well as the percentage of normal morphological spermatozoa, were significantly better in the transplant recipients than in the uremic group [59]. This indicates that successful transplantation, even with calcineurin inhibitor-based immunosuppression, results in an overall improvement in sperm function compared to the pre-transplant uremic state.

An interesting clinical observation concerns the reversibility of calcineurin inhibitors-induced sperm dysfunction following drug switching. After transplantation, the patient was treated with 10 mg per day of prednisone, 200 mg per day of CsA, and 2 mg per day of rapamycin for the first 3 months and then 10 mg per day of prednisone and 7 mg per day of rapamycin for the next 33 months. A significant improvement in semen parameters and restoration of fertility were documented after switched from rapamycin to FK506 at a dose of 5 mg/day. This suggests that the choice of immunosuppressive agent can affect sperm quality [67].

Clinical evidence indicates that, while calcineurin inhibitor-based multidrug immunosuppressive regimens are associated with acute impairment of sperm parameters post-transplantation, recovery occurs within one to two years in most recipients. Maintenance of calcineurin inhibitors levels within the therapeutic range (particularly the lower range) is associated with normal sperm parameters and long-term fertility. Although available clinical evidence does not provide convincing proof of persistent sperm DNA damage, dedicated studies of sperm DNA fragmentation, chromatin integrity, and mutational endpoints remain limited. Available paternity and pregnancy outcome data provide reassuring, although incomplete, evidence regarding reproductive safety.

## 8. Pathomechanism of Calcineurin Inhibitors in the Male Reproductive System

From the perspective of gonadotoxicity, it is important to note that calcineurin inhibition is not limited to the immune compartment. The calcineurin/NFAT pathway functions in non-immune tissues, including somatic and germ cells of the testis and epididymis, where it regulates calcium-dependent processes, mitochondrial homeostasis, sperm maturation, and the acrosome reaction [18,23,27]. Therefore, two parallel consequences of calcineurin inhibitor therapy must be distinguished: (I) an immunological consequence, involving disruption of the immune tolerance necessary for normal spermatogenesis and a shift in cytokine balance [23], and (II) a non-immunological consequence, resulting from the direct inhibition of calcineurin in spermatogenic epithelial cells, Sertoli and Leydig cells, and in maturing sperm [18,27,37].

### 8.1. Direct Cellular Targets

#### 8.1.1. Testis

In the testis, calcineurin is present in both the somatic and germinal compartments. The basic subunits (PPP3CA/PPP3R1) have been detected in Sertoli and Leydig cells, where they couple calcium signals to trophic and steroidogenic functions. In turn in the germline, the sperm-specific isoform, encoded by the PPP3CC (catalytic subunit) and PPP3R2 (regulatory subunit) was described, which is essential for normal sperm motility and fertilizing capacity [18,27]. Experimental studies revealed that calcineurin signalling has been associated with sperm maturation and the development of normal motility. Sperm-specific PPP3CC/PPP3R2 is particularly important for the function of sperm in the epididymis. Disruption to this pathway leads to reduced motility and impaired fertilisation. This has been demonstrated in PPP3CC knockout models, in which the sperm were able to penetrate the stratum granulosum and bind to the zona pellucida, but were unable to fertilise oocytes with an intact zona pellucida [18,27].

Experimental studies indicate that exposure to calcineurin inhibitors can induce structural and functional alterations to the epithelium of the seminiferous tubules. These effects may involve direct interference with calcineurin-dependent signalling in testicular cells, as well as secondary endocrine, oxidative and mitochondrial mechanisms. In rat models, CsA causes a dose- and time-dependent decrease in testis weight, as well as a reduction in the diameter of the seminiferous tubules and the thickness of the germinal epithelium. The severity of these changes ranging from mild cellular disorganization to massive degeneration of primary spermatocytes, spermatids, and Sertoli cells, accompanied by vacuolization and loss of Sertoli–germ cell junctions [12,29,31,32,33].

CsA also damages the interstitial compartment. Administration of CsA caused atrophy of Leydig cells without altering their number, as evidenced, among others findings, by a decrease in the mitochondrial compartment and the smooth endoplasmic reticulum [37]. These ultrastructural alterations are consistent with impaired steroidogenic activity. Further experimental studies indicate reduced testicular steroidogenic enzyme activity and impaired responsiveness to LH following CsA exposure [68]. Prolonged exposure to lower doses leads to interstitial fibrosis, capillary congestion, and edema [12,29,33,34].

#### 8.1.2. Epididymis

In the epididymis, the expression of pathway components shows distinct segmentation. A recent murine study provides evidence that FKBP12.6 expression and FK506 responsiveness appear to vary along the epididymis, with particularly pronounced effects observed in immature sperm during early epididymal maturation [27]. According to Meistrich’s classic data, the transition of sperm from the testis to the cauda of the epididymis takes approximately 4–10 days, and abnormalities in tail sperm appear as early as 4–5 days after administration of CsA or FK506. This locates the primary defect in the testis and the initial part of the epididymis, and not in the final place of sperm storage [27,69]. Epididymal sperm maturation, controlled almost exclusively by post-translational modifications and exosomal transfer from the epididymal epithelium, provides an additional target for calcineurin inhibitor. This is because as the epididymal epithelium is the source of regulatory proteins (including down syndrome critical region 1 (DSCR1.1) known as regulator of calcineurin 1 (RCAN1)) incorporated into sperm during transit [27,70].

### 8.2. Cellular Consequences

At the subcellular level, calcineurin inhibitor-induced toxicity in male reproductive system results from oxidative stress, mitochondrial dysfunction, apoptosis, and chronic microinflammation.

#### 8.2.1. Oxidative Stress

CsA increases the production of reactive oxygen species (ROS) in reproductive tissues, leading to membrane lipid peroxidation, protein oxidation, and DNA damage [23,32,33,35]. In the reproductive tissues of rats exposed to CsA, an increase in the levels of lipid peroxidation products (including malondialdehyde) has been reported, accompanied by a decrease in the activity of antioxidant enzymes, primarily superoxide dismutase (SOD) and glutathione peroxidase (GPx), which reduces antioxidant capacity and sensitizes sperm to oxidative stress [32,33]. However, these data should be considered a probable but not fully documented component of this pathway.

#### 8.2.2. Mitochondrial Dysfunction

Mitochondrial dysfunction serves as the link between oxidative stress, apoptosis, and sperm energy deficiency. Mitochondrial damage reduces adenosine triphosphate (ATP) synthesis and mitochondrial membrane potential (MMP), while also generating secondary ROS. In sperm, excess ROS reduces viability, motility, and MMP [23,27]. In a mouse model, inhibition of calcineurin by FK506 reverses the dephosphorylation of dynamin-like protein 1 (Drp1) at Ser637, which enhances Drp1 phosphorylation, disrupts the balance of mitochondrial fusions and fissions, and leads to mitochondrial ROS overload and a decrease in MMP. FKBP12.6 deficiency abolishes Drp1 hyperphosphorylation and partially protects immature sperm from this effect [27]. The functional link between sperm calcineurin and mitochondrial homeostasis is further supported by evidence that spermatogenesis-associated protein 33 localizes sperm-specific calcineurin to mitochondria and is required for normal sperm motility [71].

#### 8.2.3. Apoptosis

Mitochondrial damage triggers the intrinsic (mitochondrial) apoptosis pathway through membrane destabilization and the release of proapoptotic factors. Consequently, apoptosis of germ cells is observed in testis under calcineurin inhibitors [14,39]. However, it is unclear whether this pathway plays a role in the impaired fertility associated with calcineurin inhibitors in human renal transplant recipients. It should be clearly noted, however, that quantitative data regarding the Bax/Bcl-2 ratio, cytochrome c release, and the activation of caspases 9 and 3 in testis exposed to calcineurin inhibitors remain limited and are largely inferred from mitochondrial dysfunction. Their direct confirmation in this model requires further research. In turn, the role of the receptor-mediated pathway (Fas/Fas ligand) in calcineurin inhibitor-induced gonadotoxicity has not been unequivocally established and remains hypothetical.

#### 8.2.4. Inflammation

The inflammatory component may further exacerbate calcineurin inhibitor-associated reproductive injury through alterations in pro-inflammatory cytokine signaling (including tumor necrosis factor α, interleukin 1β, IFN-γ) and the mitogen-activated protein kinase and signal transducer and activator of transcription pathways, while simultaneously modulating NF-κB. Inflammation and oxidative stress may form a positive feedback loop that contributes to germ cell apoptosis, Leydig cell dysfunction, and spermatogenesis disorders [23]. However, interpretation of the direction of NF-κB changes in the testis requires caution, as the data come mainly from extragonadal models and do not allow for an unambiguous conclusion as to whether inhibition or paradoxical activation of this factor predominates in testicular tissue.

#### 8.2.5. Fibrotic and Vascular Remodeling

Interstitial fibrosis, morphologically documented as an accumulation of collagen fibers [12,14,33,34], may involve TGFβ/α-smooth muscle actin-mediated extracellular matrix remodelling. However, this mechanism has not yet been demonstrated directly in testes exposed to calcineurin inhibitors. Capillary congestion and interstitial edema further indicate disturbances in microcirculation and endothelial function. Direct data on the expression of angiogenic factors in the testis under calcineurin inhibitors treatment are scarce. However, endothelial dysfunction is a well-known post-gonadal effect of calcineurin inhibitors, co-occurring with hypertension, which indirectly supports the hypothesis of a vascular component of the gonadal damage.

### 8.3. Functional Consequences

#### 8.3.1. Impaired Steroidogenesis

A biochemically confirmed decrease in testosterone are also associated with patogenesis. A deficiency of androgens impairs the maintenance of Sertoli cell-dependent spermatogenesis, exacerbating the epithelial atrophy caused by the direct toxicity of calcineurin inhibitors [23,36]. In experimental rat models, CsA has been shown to reduce the activity of testicular cytochrome P450-dependent steroidogenic enzymes, including cholesterol side-chain cleavage and 17α-hydroxylase activities. Moreover, CsA may disrupt the HPG axis, by suppressing GnRH and gonadotropins [18,68,72].

#### 8.3.2. Altered Sertoli–Germ Cell Interaction

The loss of Sertoli–germ cell junctions may explains both the premature shedding of immature cells into the tubule lumen and the arrest of spermatogenesis [32,33]. In the long-term model, atrophy was accompanied by a reduction in the number of mature spermatids and ultrastructural changes in Sertoli cell organelles, indicating that even doses that do not cause acute degeneration impair the trophic function of Sertoli cells toward the germline [34].

#### 8.3.3. Impaired Epididymal Maturation

Experimental models revealed that the epididymal epithelium may exhibit changes as a result of FK506 toxicity [27]. Through exosomal transfer and post-translational modifications, it delivers DSCR1.1 (RCAN1) to sperm cells. A recent mouse study demonstrated that this protein is an endogenous calcineurin inhibitor. Its overexpression enhances the inhibition of sperm-specific calcineurin and reduces the expression of PPP3CC/PPP3R2 and synaptosomal-associated protein 25 (SNAP-25), a soluble N-ethylmaleimide-sensitive factor attachment protein receptor essential for Ca^2+^-dependent acrosomal exocytosis [27,70]. Consequently, both motility (through dysregulation of Ca^2+^ signaling and mitochondrial homeostasis) and the ability to undergo the acrosome reaction upon binding to the zona pellucida are impaired, which explains the decrease in fertilization capacity regardless of sperm count [18,27]. Changes in epididymal fluid composition and impaired sperm transport may confirm impaired epididymal homeostasis under the influence of calcineurin inhibitors [31,36,44].

#### 8.3.4. Altered Semen Parameters and Fertility

In clinical studies of men who had undergone kidney transplantation, sperm motility proved to be the parameter most severely and earliest affected, with marked deficits in both fertile and infertile groups. However, sperm count showed greater variability and, in some patients, remained within WHO normal limits despite the absence of offspring [56]. A dose-dependent analysis showed that at low CsA doses semen parameters did not differ from those of controls, whereas at the higher doses abnormalities in motility and morphology appeared [64,66]. DNA integrity and chromatin condensation under the influence of calcineurin inhibitors have not been unequivocally demonstrated as impaired in the available clinical studies. Furthermore, classical analyses have not confirmed the genotoxicity of CsA in sperm, and the observed congenital anomalies in offspring did not occur, suggesting that the dominant mechanism of infertility is a functional defect (motility, acrosome reaction) rather than structural damage to the sperm genome [56,73]. Experimental evidence suggests that fertility is impaired by both motility defects and impaired acrosome exocytosis, which are caused by reduced SNAP-25 levels and Ca^2+^ dysregulation, a mechanism that has been demonstrated for FK506 [27]. In men who have undergone kidney transplantation, despite the use of calcineurin inhibitors, fertility is possible and, after a period of approximately 15–24 months, with good graft function and low trough drug concentrations, it approaches that of the general population, indicating that the functional defect is partially reversible [56,59,64,73].

### 8.4. Confounders

The main controversies concern inconsistencies in results across studies, which most often stem from differences in species and strain, dose, route of administration, animal age (neonatal and prepubertal models versus adult models), and duration of exposure.

Importantly, comparisons based solely on administered dose may be misleading, as pharmacokinetic profiles, systemic exposure, tissue distribution, and drug metabolism differ substantially between species and routes of administration. The timing of exposure is also critical, as developing and sexually mature animals may differ considerably in their susceptibility to calcineurin inhibitors-induced reproductive injury. Likewise, treatment duration and the timing of outcome assessment may influence the phenotype detected, with prolonged exposure potentially resulting in structural remodeling in addition to early biochemical or functional alterations.

Transplant-specific confounders significantly complicate clinical interpretation. Chronic kidney disease itself impairs gonadal function through various mechanisms, such as uremic suppression of GnRH and gonadotropins. The duration and intensity of pre-transplantation dialysis therapy are independently associated with persistent hypogonadism post-transplantation. Glucocorticoid exposure suppresses the HPG axis in a dose-dependent manner by inhibiting ACTH secretion. These effects are clearly separable from calcineurin inhibitor mechanisms. The status of renal allograft function correlates directly with hormonal recovery, suggesting that improvement in renal clearance (rather than a reduction in calcineurin inhibitor exposure alone) drives the normalisation of reproductive parameters in many recipients. Failure to account for these variables when assessing calcineurin inhibitor gonadotoxicity may result in reproductive dysfunction being misattributed to direct calcineurin inhibition when the underlying cause is a transplantation-related complication.

## 9. Differences Between Cyclosporine A and Tacrolimus

Although both drugs inhibit calcineurin via immunophilins, their gonadotoxicity profiles differ both quantitatively and qualitatively. CsA forms a complex with cyclophilins, while FK506 forms a complex with FKBP12/FKBP12.6. The FK506–FKBP complex inhibits calcineurin more potently than the CsA–cyclophilin complex, which in T cells results in stronger suppression of NFAT-dependent transcription [74,75]. In reproductive tissues, this difference is further amplified by the mechanism described in mice. FK506, unlike CsA, induces overexpression of DSCR1.1 and degradation of sperm-specific PPP3CC/PPP3R2, as well as reduced expression of SNAP-25, with these effects being dependent on FKBP12.6 and, in part, on FKBP12 [27]. This means that the inhibition of calcineurin by FK506 in sperm is enhanced by a feedback loop involving an endogenous inhibitor and the loss of the enzyme. This provides a molecular explanation for the greater potency of FK506 in terms of sperm maturation and motility. However, in classical histological models of the testis, FK506 is sometimes milder than CsA [12,27].

## 10. Materials and Methods

A comprehensive literature search was performed in PubMed (MEDLINE) for studies published between January 1984 and July 2026. The final search was conducted on 31 July 2026. The start date was chosen to coincide with the introduction of CsA into widespread clinical use in kidney transplantation.

The search combined Medical Subject Headings and free-text title/abstract terms into two conceptual categories: (1) exposure (calcineurin inhibitors) and (2) outcome/population (male reproductive function and kidney transplantation). The following terms were used: ‘Cyclosporine’, ‘Cyclosporin’, ‘CsA’, ‘Tacrolimus’, ‘FK506’, ‘calcineurin inhibitors’, ‘CNI’ and ‘testis’, ‘male gonad’, ‘epididymis’, ‘prostate’, ‘semen’, ‘hypothalamic–pituitary–gonadal axis’, ‘HPG axis’, ‘fertility’, ‘kidney transplantation’, ‘renal transplantation’, ‘kidney transplant’, ‘renal transplant’. Terms within each category were combined using ‘OR’, and the two categories were combined using ‘AND’. No language filter was applied at the search stage.

The inclusion criteria were: (1) Original in vivo experimental animal studies; in vitro studies; and clinical studies (randomised controlled trials; cohort studies; cross-sectional studies; case–control studies; case series; and case reports); (2) Evaluation of CsA and/or FK506 as monotherapy; combination therapy; or a comparative regimen; and (3) Reporting of outcomes related to the male reproductive system. These include testicular histology; spermatogenesis/semen parameters; the epididymis; the prostate; fertility; and HPG axis hormones. Exclusion criteria were: (1) reviews, editorials and conference abstracts without sufficient data; (2) studies that did not specify the immunosuppressive agent used; and (3) studies that were limited to female reproduction or non-calcineurin inhibitors immunosuppression.

All retrieved records were screened by title and abstract, followed by a full-text assessment to determine their eligibility. The reference lists of the full-text articles included and relevant reviews were also hand-searched for eligible studies. For non-English articles, only the English abstract was used for data extraction.

After the full-text assessment, a total of 41 studies were included in this review: 21 in vivo experimental studies, 19 clinical studies, and 1 in vitro study. The experimental studies comprised 9 studies on CsA monotherapy, 4 studies on FK506 monotherapy, 2 studies on both CsA and FK506 monotherapy, and 6 studies on FK506 or CsA in multidrug therapy. The clinical studies comprised 1 study on CsA monotherapy, 0 studies on FK506 monotherapy, and 18 studies on FK506 or CsA in multidrug therapy. The in vitro studies comprised 1 study on CsA monotherapy.

## 11. Conclusions

In experimental animal and in vitro models, the proposed mechanisms of calcineurin inhibitor-associated toxicity in the male reproductive system primarily indicate the following: (1) potential interference with calcineurin-dependent signalling pathways in testicular somatic cells, germ cells and maturing spermatozoa, (2) induction of oxidative stress, characterised in rodent studies by a decrease in SOD/GPx and an increase in lipid peroxidation, (3) mitochondrial dysfunction with a decrease in mitochondrial membrane potential and ATP, (4) interstitial alterations, where high-dose CsA in rodents appears to induce Leydig cell atrophy and reduced steroidogenesis, (5) disorganisation of the seminiferous epithelium with destabilised Sertoli–germ cell junctions, and (6) impaired post-testicular sperm maturation, where murine data specifically implicate an FKBP12.6/RCAN1/SNAP-25-dependent cascade in defective acrosomal exocytosis and motility following FK506 exposure (Figure 2).

The observed discrepancies between CsA and FK506, which manifest as more pronounced histological disruption in CsA-treated rodents and predominant molecular impairment of sperm functional competence in FK506-treated rodents, likely reflect divergent tissue sensitivities and distinct immunophilin-binding profiles.

Importantly, the direct translation of these preclinical observations to kidney transplant recipients remains problematic. While experimental models usually assess high-dose monotherapy, clinical outcomes in patients occur within the context of multidrug immunosuppression. These outcomes are influenced by factors such as the severity of pre-existing chronic kidney disease, the duration of dialysis, graft clearance and the administration of glucocorticoids. Rather than causing irreversible reproductive failure, clinical evidence suggests that successful renal engraftment often promotes partial recovery of the HPG axis and spermatogenesis within one to two years. Consequently, the current evidence base does not support classifying calcineurin inhibitors as unequivocally gonadotoxic at target trough levels in human therapeutics, and well-controlled, prospective clinical studies are required to distinguish drug-specific effects from systemic, transplant-related confounders.

## 12. Limitations and Research Gaps

The research limitations include a lack of clinical histological studies of the testes and epididymis, the absence of standardized measurements of sperm DNA fragmentation and chromatin condensation under the influence of calcineurin inhibitors, incomplete characterization of apoptotic and autophagy pathways in the gonads, and insufficient knowledge regarding the direct effects on the blood-testis barrier.

A fundamental limitation of the current evidence base is the significant translational gap between experimental rodent models and the clinical reality of male kidney transplant recipients. Most mechanistic data on calcineurin inhibitor-induced gonadotoxicity has been obtained from healthy animals or non-transplant models subjected to calcineurin inhibitor monotherapy. However, critical interspecies differences in reproductive physiology, such as the shorter duration of spermatogenesis in rodents compared to humans, distinct pharmacokinetic profiles, and variations in the expression and tissue distribution of calcineurin isoforms and immunophilins, hamper direct extrapolation of these findings to humans. Furthermore, experimental models often fail to replicate the complex uremic environment, systemic inflammation and drug interactions that are typical of end-stage renal disease and post-transplant immunosuppression. Consequently, while animal studies provide essential proof-of-concept evidence for potential mechanisms of injury, they cannot definitively establish the magnitude and reversibility of these effects in human transplants.

Most clinical studies critically lack adequate stratification and adjustment for transplant-related confounding factors, including baseline chronic kidney disease stage, cumulative dialysis exposure, current allograft function, cumulative glucocorticoid dose and concomitant immunosuppressive agents. Prospective studies that incorporate a detailed assessment of these variables and use multivariate analysis are essential in order to isolate the direct gonadotoxic effects of calcineurin inhibitors from the broader impact of end-stage renal disease and its treatment on male reproductive function.

It is worth noting that calcineurin inhibitors can have clinically significant metabolic effects that may indirectly affect male reproductive function [9]. Both CsA and FK506 may impair glucose homeostasis by reducing pancreatic β-cell insulin secretion, with tacrolimus generally being associated with a higher risk of post-transplantation diabetes mellitus. Conversely, CsA is more consistently associated with dyslipidaemia, including increased total and low-density lipoprotein cholesterol, and in some studies, increased triglyceride concentrations. These effects are modified by glucocorticoid exposure, adiposity, renal allograft function and concomitant immunosuppressive therapy. Hyperglycaemia, insulin resistance and dyslipidaemia may contribute to oxidative stress, endothelial dysfunction and hypogonadism. Therefore, they should be considered potential indirect mediators of reproductive impairment [76,77,78].

## Figures and Tables

**Figure 1:**
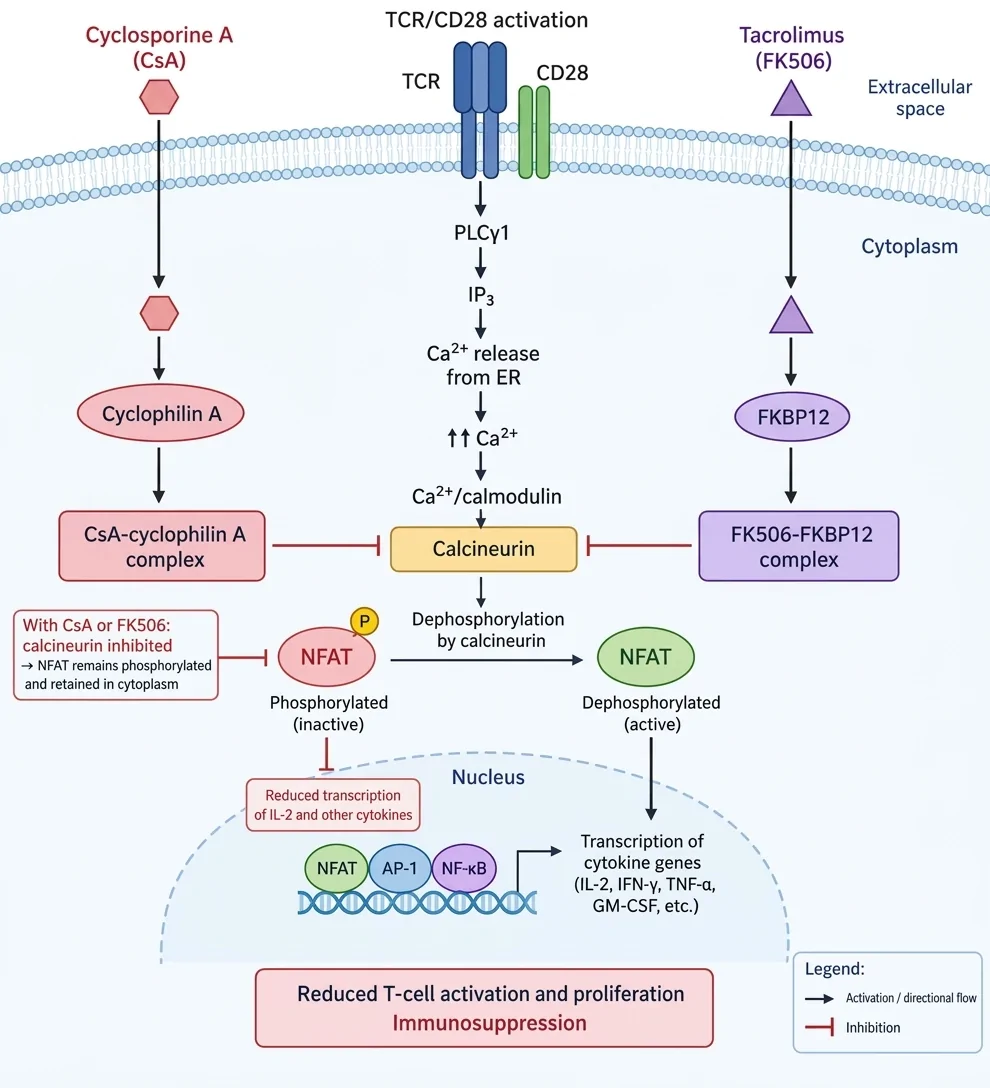
**The mechanism of the immunosuppressive action of cyclosporine A (CsA) and tacrolimus (FK506) via the calcineurin–nuclear factor of activated T cells (NFAT) pathway**. Activation of the T cell receptor (TCR/CD28) leads to activation of phospholipase C gamma 1 (PLCγ1), the formation of inositol trisphosphate (IP_3_), and the release of Ca^2+^ from the endoplasmic reticulum (ER). The increase in Ca^2+^ concentration enables Ca^2+^/calmodulin-dependent activation of calcineurin, which dephosphorylates NFAT, allowing it to translocate to the nucleus and interact with other transcription factors. CsA forms a complex with cyclophilin A, while FK506 forms a complex with FK506-binding protein 12 (FKBP12). These complexes inhibit calcineurin activity, preventing the dephosphorylation and nuclear translocation of NFAT. Consequently, transcription of cytokine genes is reduced, as is the activation and proliferation of T lymphocytes, leading to immunosuppression. Abbreviations: AP-1—activator protein 1; CsA—cyclosporine A; ER—endoplasmic reticulum; FK506—tacrolimus; FKBP12—FK506-binding protein 12; GM-CSF—granulocyte-macrophage colony-stimulating factor; IFN-γ—interferon γ; IL-2—interleukin 2; IP_3_—inositol trisphosphate; NFAT—calcineurin–nuclear factor of activated T cells; NF-κB—nuclear factor kappa-light-chain-enhancer of activated B cells; PLCγ1—phospholipase C gamma 1; TCR/CD28—T cell receptor; TNF-α,—tumor necrosis factor α.

**Figure 2:**
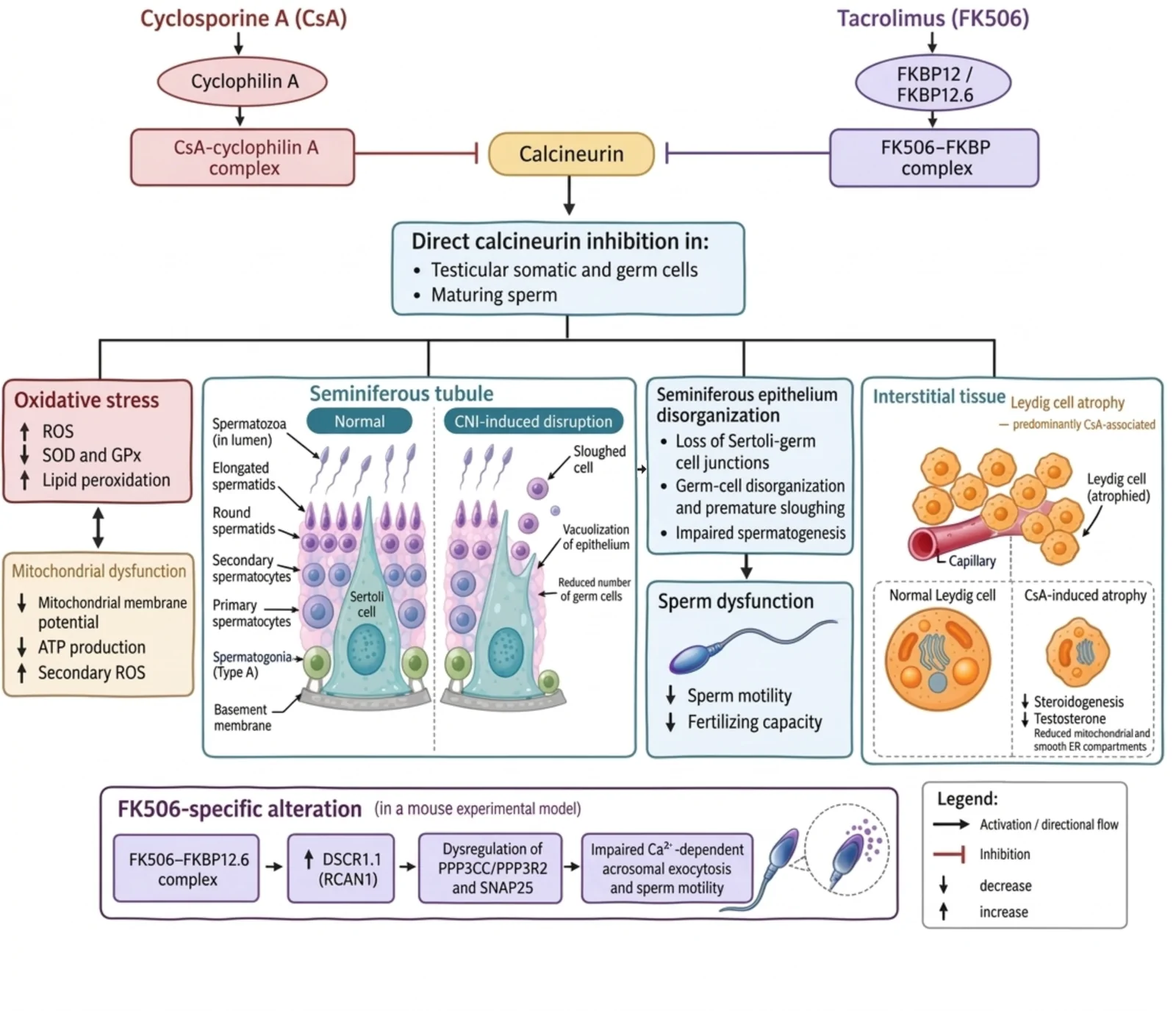
**Proposed mechanisms of calcineurin inhibitor-induced gonadotoxicity.** Cyclosporine A (CsA) and tacrolimus (FK506) bind to cyclophilin A and FKBP12/FKBP12.6, respectively. These complexes inhibit calcineurin in testicular somatic and germ cells, as well as in maturing sperm. Inhibition of calcineurin is associated with oxidative stress, which is characterised by increased reactive oxygen species, decreased superoxide dismutase (SOD) and glutathione peroxidase (GPx) activity, and increased lipid peroxidation. Mitochondrial dysfunction can further decrease mitochondrial membrane potential and ATP production, amplifying oxidative injury. CsA-associated Leydig cell atrophy results in impaired steroidogenesis and reduced testosterone production. Disruption of Sertoli–germ cell junctions in the seminiferous epithelium promotes germ-cell disorganisation, premature sloughing, and impaired spermatogenesis. In sperm, calcineurin inhibition reduces motility and impairs the acrosome reaction. In experimental models, sperm dysfunction associated with FK506 is further amplified through an FKBP12.6-dependent DSCR1.1/RCAN1–PPP3CC/PPP3R2–SNAP25 pathway. This results in defective Ca^2+^-dependent acrosomal exocytosis and reduced fertilising capacity. Solid arrows indicate activation or directional flow; red blunt-ended lines indicate inhibition. Abbreviations: ATP—adenosine triphosphate; CNI—calcineurin inhibitor; CsA—cyclosporine A; DSCR1.1—down syndrome critical region 1; FK506—tacrolimus; FKBP—FK506-binding protein; GPx—glutathione peroxidase; PPP3CC—protein phosphatase 3 catalytic subunit gamma; PPP3R2—protein phosphatase 3 regulatory subunit B beta; RCAN1—regulator of calcineurin 1; ROS—reactive oxygen species; SNAP-25—synaptosomal-associated protein 25; SOD—superoxide dismutase.

## Data Availability

No new data were created or analyzed in this study. Data sharing is not applicable to this article.

## Abbreviations

The following abbreviations are used in this manuscript:

CsA	cyclosporine A
FK506	tacrolimus
HPG	hypothalamic–pituitary–gonadal
PP2B	protein phosphatase 2B
PPP3CA	protein phosphatase 3 catalytic subunit alpha
PPP3CB	protein phosphatase 3 catalytic subunit beta
PPP3CC	protein phosphatase 3 catalytic subunit gamma
PPP3R1	protein phosphatase 3 regulatory subunit B alpha
PPP3R2	protein phosphatase 3 regulatory subunit B beta
NFAT	nuclear factor of activated T cells
IL-2	interleukin 2
IL-4	interleukin 4
TGFβ	transforming growth factor β
FKBP	FK506-binding protein
IFN-γ	interferon γ
NF-κB	nuclear factor kappa-light-chain-enhancer of activated B cells
ETS	E twenty-six
Elk-1	ETS-like transcription factor 1
AZA	azathioprine
MMF	mycophenolate mofetil
LH	luteinising hormone
FSH	follicle-stimulating hormone
hCG	human chorionic gonadotropin
DHEAS	dehydroepiandrosterone sulfate
SHBG	sex hormone-binding globulin
GnRH	gonadotrophin-releasing hormone
VSL	straight-line velocity
DSCR1.1	down syndrome critical region 1
RCAN1	regulator of calcineurin 1
ROS	reactive oxygen species
SOD	superoxide dismutase
GPx	glutathione peroxidase
ATP	adenosine triphosphate
MMP	mitochondrial membrane potential
Drp1	dynamin-like protein 1
WHO	World Health Organization
SNAP-25	synaptosomal-associated protein 25
